# Measuring osteotomy wedge angle is more important than measuring wedge height in open wedge osteotomies around the knee in preoperative planning

**DOI:** 10.1002/ksa.12609

**Published:** 2025-02-12

**Authors:** Julius Watrinet, Johannes Schlaich, Romed Vieider, Marco‐Christopher Rupp, Julian Mehl, Sebastian Siebenlist, Armin Runer

**Affiliations:** ^1^ Department of Orthopaedic Sports Medicine, School of Medicine Technical University Munich Munich Germany

**Keywords:** accuracy, DFO, hinge point, HTO, osteotomy, planning

## Abstract

**Purpose:**

Preoperative planning for medial open wedge high tibial osteotomies (HTOs) and lateral open wedge distal femur osteotomies (DFOs) commonly uses wedge height to guide accurate correction. However, it is unclear if this parameter is influenced by intraoperative variations in osteotomy entry point or length. This study hypothesized that wedge angle remains constant during planning, while wedge height varies depending on hinge or entry points.

**Methods:**

Whole leg radiographs of 40 patients who underwent HTO or DFO (2018–2024) were analysed using digital planning software. For each HTO and DFO case, 27 and 21 osteotomy variants were created, respectively, by altering entry and hinge points, resulting in 960 simulations. Wedge angle, wedge height and osteotomy depth were measured for each variant. Correlations and regression analyses assessed the relationships among these variables, and a mathematical formula was developed to predict wedge height from wedge angle and osteotomy depth.

**Results:**

Wedge angle remained consistent across variants (mean deviation: 0.1 ± 0.1°), while wedge height showed variability (mean deviation: 0.7 ± 0.5 mm) influenced by entry and hinge points. Significant correlations were found between wedge height and opening angle (*R* = 0.83, *p* < 0.001) and osteotomy depth (*R* = 0.60, *p* < 0.001). Predicted wedge height closely matched actual values (*R* = 0.998, *p* < 0.001), with minimal error (−0.01 ± 0.1°).

**Conclusion:**

This study highlights that relying solely on wedge height for osteotomy planning in HTO and DFO is insufficient due to variations in entry and hinge points. The hinge angle proved to be the most reliable parameter. Intraoperative osteotomy depth measurements can help adjust wedge height for accurate limb alignment when deviations occur.

**Level of Evidence:**

Level V, simulation study.

**Abbreviations**
BMI: body mass index
DFO: distal femur osteotomy
FCW: femur condyle width
FL: femur length
HTO: high tibial osteotomy
IQR: interquartile range
JLCA: joint line convergence angle
mFA‐mTA: mechanical femorotibial angle
mLDFA: mechanical lateral distal femur angle
MPTA: medial proximal tibia angle
SD: standard deviation
TL: tibial length
TPW: tibial plateau width
WBL: weight‐bearing line

## INTRODUCTION

Mechanical alignment correction through osteotomy is an effective joint‐preserving treatment for unicompartmental knee joint degeneration relieving pressure off the affected compartment [3, 4, 5, 10, 28, 30, 34, 36, 38, 43]. Evidence‐based indications include meniscus replacement [20, 21], cartilage regenerative surgery [2, 7] or anterior cruciate ligament deficiency [13], where osteotomy improves biomechanical load distribution. Successful osteotomy requires precise preoperative planning.

Currently, preoperative planning is mainly performed on two‐dimensional weight‐bearing lower limb radiographs [24, 32, 37], with methods based on Paley's principle and frameworks by Miniaci, Jakob, and Strecker [24, 32, 37]. These methods have evolved from printed 2D radiographs to advanced digital techniques [11], with ongoing efforts to incorporate artificial intelligence [25, 41].

Despite advancements, postoperative limb alignment often deviates from preoperative plans, with a reported accuracy of 1.5° (0.5–2.4°) for the mechanical femorotibial angle (mFA‐mTA) [1]. This inaccuracy may be based result from reliance on wedge height measurements, which, while feasible intraoperatively, may be influenced by entry and hinge points [6, 35, 41].

Measuring wedge height is straightforward intraoperatively, but its sensitivity to the osteotomy entry point and hinge location remains debated [15, 22, 23, 26, 27, 40, 42]. Variations in these points compared to preoperative plans might explain inaccuracies in postoperative alignment correction. The purpose of this simulation study was to investigate the impact of the selection of osteotomy entry and hinge point on typical preoperative planning parameters, such as wedge height, wedge angle and osteotomy depth in medial open wedge high tibial osteotomy (HTO) and lateral open wedge distal femur osteotomy (DFO). Additionally, the study aimed to determine whether the wedge angle provides superior reliability in preoperative limb alignment correction compared to wedge height. The hypothesis proposed that while the wedge angle remains a fixed parameter during preoperative planning, the wedge height can vary significantly based on the position of the hinge or entry point.

## METHODS

### Data collection

**Figure 1:**
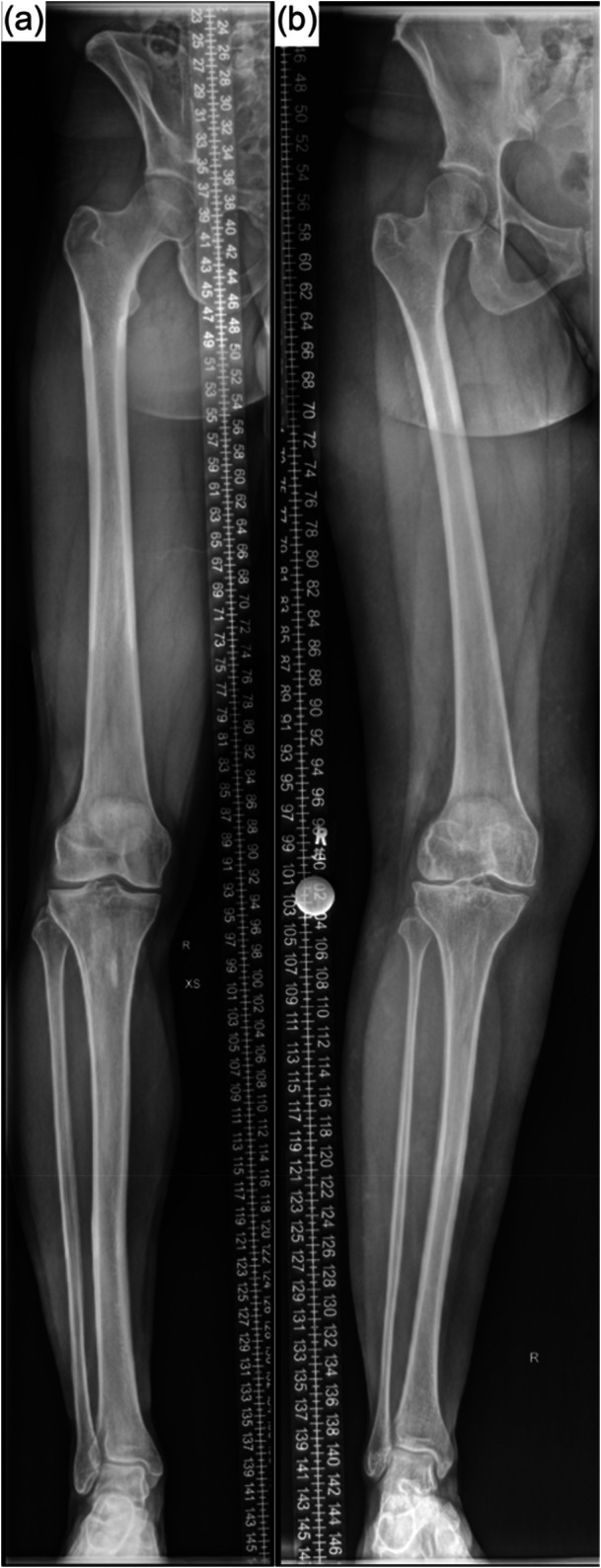
Preoperative whole leg radiographs taken in the knee forward position. (a) A preoperative genu varum while (b) shows a genu valgum.

This retrospective simulation study analysed whole leg radiographs which were taken in case of genu varum (HTO, *n* = 20) or genu valgum (DFO, *n* = 20) between September 2018 and March 2024 (Figure 1). Only patients with sufficient quality of radiographs (complete depiction of anatomical landmarks, no malrotation resulting in an obscured knee joint space, meeting the criteria for knee‐forward positioning [32], and no extension deficit exceeding 5° on clinical examination), and age between 16 and 50 years were included.

### Planning

Full weight‐bearing long leg radiographs in the knee forward position were taken using a steel reference ball with a 25 mm diameter positioned close to the knee for calibration. Initially, lower limb alignment was assessed utilizing the mediCAD® preoperative planning software v5.1 (Hectec). Osteotomies were planned according to the Miniaci method by a single rater [24]. Different osteotomy variants for each HTO and DFO were created by combining different entry and hinge points resulting in a total of 960 variants. By utilizing coordinate systems during the planning process, comparability among different patients was ensured.

**Figure 2:**
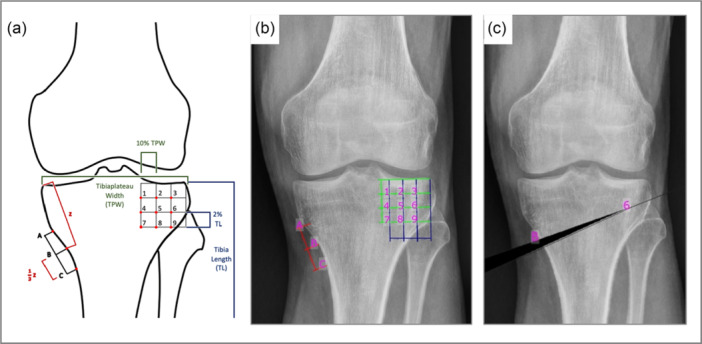
Medial open wedge proximal tibial osteotomy is planned following lower limb deformity analysis using the mediCAD® software. Per case, 27 osteotomy variants were planned utilizing three osteotomy entry points and nine hinge points (A–C). Osteotomy entry points as well as hinge points were determined in a standardized fashion in a coordinate system based on anatomic landmarks (a).

Each HTO osteotomy variant was planned with three different entry points and nine different hinge points (Figure 2), which were aligned using a standardized coordinate system. This system was defined by relative distances measured on each radiograph individually as demonstrated in the figure. Each patient underwent planning for 27 HTO osteotomies with different entry points and hinge points, resulting in a postoperative weight‐bearing line (WBL) to 55.0% of the tibial plateau's width, measured from the medial edge plateau [9, 16]. Osteotomy B6 was identified as the optimal hinge position [27].

**Figure 3:**
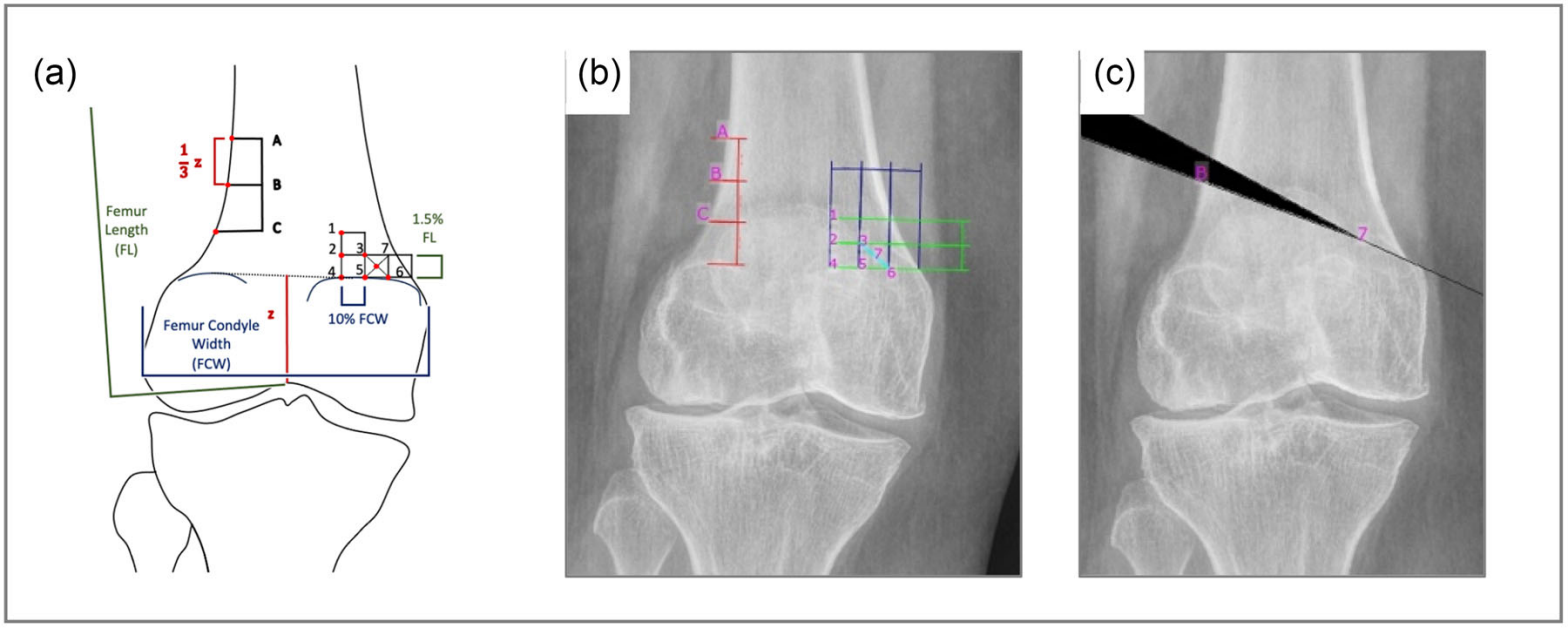
Lateral open wedge distal femur osteotomy is planned after initial lower limb deformity analysis using the mediCAD® software. Twenty‐one osteotomy variants were planned utilizing three osteotomy entry points and seven hinge points (A–C). Points were determined standardized on a coordinate system based on anatomic landmarks (a).

Twenty‐one variations of DFO, each combining a different hinge and entry point, were planned to adjust the WBL to align with 50.0% of the tibial plateau's width, measured from the medial edge of the plateau (Figure 3). Conversely, a standardized coordinate system was defined to determine hinge and entry points (Figure 3). Osteotomy B7 was considered the optimal hinge position [42].

Of each planned osteotomy, the osteotomy depth and wedge height were measured manually. The widths of the tibia and femur were measured at their widest diameters, parallel to the tibial or femoral joint line. The correction angle, as well as the mechanical lateral distal femur angle (mLDFA), medial proximal tibia angle (MPTA), joint line convergence angle (JLCA) and mFA‐mTA, were calculated using the mediCAD® software.

Wedgeheight=2*(sin(0.5*α)*osteotomydepth).

### Mathematical prediction of wedge height

**Figure 4:**
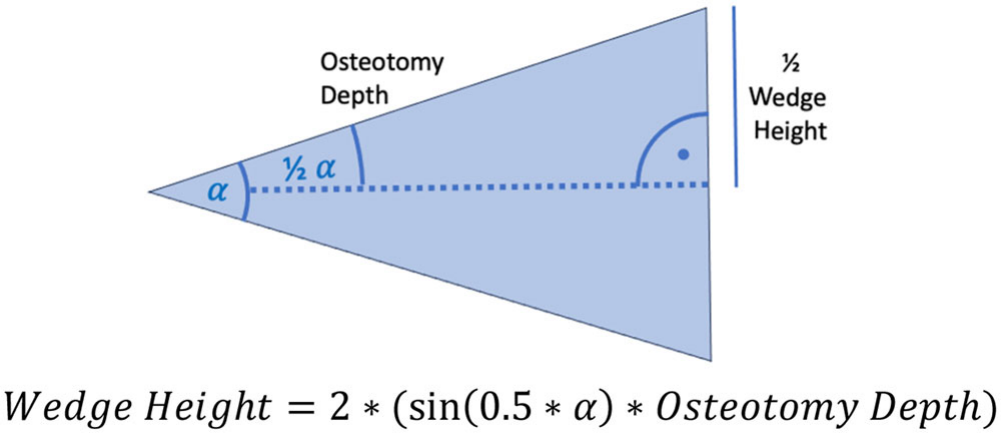
The osteotomy wedge is an isosceles triangle consisting of two identical congruent right‐angled triangles. Using the sine function, the wedge height can be calculated as displayed.

The wedge height was calculated using a formula based on the correction angle and osteotomy depth. In opening osteotomies, the wedge forms an isosceles triangle comprising two congruent right‐angled triangles, with the hypotenuses representing the osteotomy depth and the alpha angle being half the opening angle. Wedge height, calculated using the sine function, is the sum of the opposite sides relative to the hinge point (Figure 4). Example calculations are in Table 1. The wedge height was both manually measured and mathematically calculated, and deviations were determined.

**Table 1:** Predicted values of the wedge height depending on the osteotomy depth and wedge angle.^a^

		Osteotomy depth (mm)									
		30	35	40	45	50	55	60	65	70	75
Wedge angle (°)	3	1.6	1.8	2.1	2.4	2.6	2.9	3.1	3.4	3.7	3.9
	4	2.1	2.4	2.8	3.1	3.5	3.8	4.2	4.5	4.9	5.2
	5	2.6	3.1	3.5	3.9	4.4	4.8	5.2	5.7	6.1	6.5
	6	3.1	3.7	4.2	4.7	5.2	5.8	6.3	6.8	7.3	7.9
	7	3.7	4.3	4.9	5.5	6.1	6.7	7.3	7.9	8.5	9.2
	8	4.2	4.9	5.6	6.3	7.0	7.7	8.4	9.1	9.8	10.5
	9	4.7	5.5	6.3	7.1	7.8	8.6	9.4	10.2	11.0	11.8
	10	5.2	6.1	7.0	7.8	8.7	9.6	10.5	11.3	12.2	13.1

^a^ Values are calculated based on a formular [wedge height = 2 * (sin (0.5 * α) * osteotomy depth)]. Wedge height values were continuously coloured from blue (low) to white to red (high).

### Sample size calculation

The sample size was calculated using G*Power 3.1.9.6 software. The calculation was based on the observed positive correlation (*r*² = 0.6) between the mathematically determined wedge height and osteotomy depth as presented in Table 1. To achieve a statistical power of 95% with an effect size (Cohen's *d*) of 0.6 and an *α* level of 0.05, a minimum of 13 participants was required for each group to detect a significant correlation. The study was approved by the ethical committee of the Technical University Munich (2024‐318‐S‐CB).

### Statistical analysis

The normality of continuous variables was assessed using the Kolmogorov–Smirnov test. Normally distributed variables were presented as means with standard deviations, while non‐normally distributed variables were presented as medians with interquartile ranges (IQRs). The correlation between continuous variables was analysed via Pearson's chi‐square. A multiple linear regression, including wedge height, wedge angle, osteotomy depth and tibial or femoral width, was used to determine the predictive value of the osteotomy simulation‐specific variables in relation to wedge height. Findings with *p* < 0.05 were considered statistically significant. Statistical analyses were performed using R version 2023.03.1 (Copyright 2016, The R Foundation).

## RESULTS

Patient demographics, preplanning measurements and postplanning measurements for HTO and DFO, are presented in Table 2. Tables A1 and A2 display the median wedge height as well as osteotomy depth for each of the different planned osteotomies.

**Table 2:** Patient characteristics and planning results by osteotomy.^a^

	DFO (*n* = 20) median [*Q*1–*Q*3]	HTO (*n* = 20) median [*Q*1–*Q*3]
Female sex	50%	50%
Age (years)	30.0 [23.8–34.3]	38.0 [24.8–41.3]
BMI (kg/m^2^)	30.4 [25.6–37.0]	25.1 [22.3–27.2]
Preplanning measurements		
mLDFA	84.3 [81.8–86.2]	88.8 [85.6–91.8]
MPTA	89.5 [85.8–92.1]	85.35 [81.7–87.4]
JLCA	1.3 [0–2.7]	1.5 [0.1–3.2]
mFA‐mTA (°)	5.7 [3.6–11.2]	−4.3 [−7.1 to −2.7]
Femur width/tibia width	79.6 [61.4–92.8]	72.5 [67.0–88.0]
Measurements after planned correction of WBL to 50% (DFO) or 55% (HTO)		
mLDFA	89.5 [85.2–92.3]	88.8 [85.6–91.7]
MPTA	89.5 [85.8–92.1]	91.0 [88.5–94.2]
JLCA	1.3 [0–2.7]	1.5 [0.1–3.2]
mFA‐mTA (°)	0.4 [0.1–0.7]	1.3 [1–2]

Abbreviations: BMI, body mass index; DFO, distal femur osteotomy; HTO, medial open wedge high tibial osteotomy; JLCA, joint line conversion angle; mFA‐mTA, mechanical femorotibial angle; mLDFA, mechanical lateral distal femur angle; MPTA, medial proximal tibia angle; WBL, weight‐bearing line.

^a^ Values are presented as no. (%) or median and the interquartile range.

The wedge angle in different osteotomy variants remained constant in each case with a mean difference of 0.1 ± 0.1° (range 0.0–0.4°), while the wedge height exhibited a variability of 0.7 ± 0.5 mm (range 0.0–2.6 mm). Similarly, there was no difference of the corrected MPTA in HTO (0.0 ± 0.0°) and corrected mLDFA in DFO (0.0 ± 0.0°) in each planning case.

The wedge heights obtained by either calculation or manual measurements were highly correlated (*R* = 0.998, *p* < 0.001), with a mean difference of −0.01 ± 0.1 mm.

**Figure 5:**
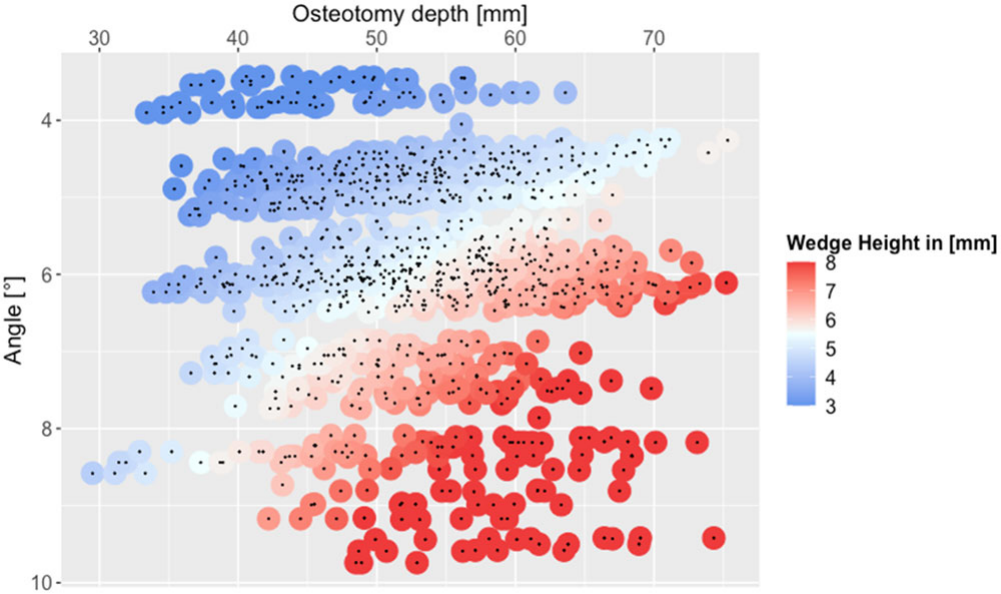
A data point for each osteotomy was created depending on the value for the osteotomy depth and the wedge angle. Points were continuously coloured from blue (low) to white to red (high) based on the wedge height value.

Significant correlations were observed between wedge height and osteotomy depth (*p* < 0.001, *R* = 0.60, Figure 5), as well as between wedge height and opening angle (*p* < 0.001, *R* = 0.83). Additionally, wedge height demonstrated significant correlations with tibial width (*p* < 0.001, *R* = 0.40) and femoral width (*p* < 0.001, *R* = 0.24).

In the multiple linear regression analysis, several significant influencing factors on wedge height were identified. Both opening angle (estimate = 0.9, *p* < 0.001) and osteotomy depth showed a significant positive influence (estimate = 0.1, *p* < 0.001) on wedge height. Conversely, tibial and femoral width did not significantly impact wedge height (*p* = 0.13).

## DISCUSSION

This study identified the wedge angle as a more reliable parameter than wedge height in planning open wedge osteotomies for HTO and DFO. Relying solely on wedge height can be problematic, as variations in the planned osteotomy entry and hinge points may compromise the accuracy of postoperative correction. The observed positive correlation between wedge height, osteotomy angle and depth supports this finding, as the calculation of wedge height is fundamentally based on the ray theorem, which incorporates both osteotomy depth and wedge angle. These results have important clinical implications. Any deviation in entry or hinge points affects wedge height and osteotomy depth, emphasizing the need for precise adherence to preoperative planning. To minimize errors, surgeons are encouraged to use intraoperative fluoroscopy to verify hinge positioning and osteotomy depth. Importantly, the study found that the wedge angle remained consistent across cases where preoperative planning aimed for identical limb axis corrections. This consistency underscores the value of wedge angle measurement as a reliable intraoperative tool for verifying alignment. This study highlights the need to translate the two‐dimensional wedge angle into a three‐dimensional operative setting for open wedge osteotomies. This is typically achieved by converting the planned wedge angle into the corresponding wedge height required for the desired correction, a process first introduced by Hernigou's correction table [11]. Intraoperative alterations to the osteotomy entry point and hinge point can lead to unintended changes in the osteotomy depth and wedge height, resulting in undesired postoperative outcomes.

A similar study found no significant changes in wedge height with varying osteotomy entry points [23]. This finding does not align with our results, as different starting points alter osteotomy depth and wedge height. Differences may be attributed to small sample sizes. Limited variability, or the comparisons of absolute values without considering the association between osteotomy depth and mediolateral diameter.

Experimental findings in cadaveric specimens further support the dependency between hinge position and wedge height, showing that altering the hinge position while maintaining a constant wedge height significantly affects the MPTA in HTO [15].

Based on the study's results, surgeons must closely align preoperative planning with intraoperative execution. Accurate placement of the entry and hinge points during surgery is critical for achieving proper limb alignment. In cases of deviations, surgeons can rely on the correction table (Table 1) and measure osteotomy depth and recalculate wedge height, maintaining the planned correction angle. This requires fluoroscopy‐guided measurements orthogonal to the hinge axis, with the knee positioned in the anterior–posterior direction and aligned horizontally. Maintaining the hinge axis in the anteroposterior plane is essential to avoid unintended alterations in the sagittal plane, as hinge position significantly affects the posterior tibial slope in HTO [12, 14, 39]. Bone loss from the saw blade's thickness should also be accounted for to enhance accuracy. Minor deviations from preoperative plans can significantly impact limb alignment, knee functionality and arthrosis progression [8, 19].

Techniques such as intraoperative measurement of wedge height using metal probes, calipers, or fluoroscopy, or assessing alignment through radiographs, alignment rods, calibrated grids or cable methods, can verify corrections effectively [17, 33, 44]. Although navigated osteotomies have been introduced, they have not demonstrated superior accuracy over traditional methods [29].

### Limitations

This simulation study acknowledges several limitations. Hinge points that do not conform to Paley's principles of deformity correction were included to explore the mathematical relationships involved, despite their impracticality [18, 31]. The performance of osteotomies is inherently limited by various factors, including bony characteristics, soft tissue structures, and the design and sizing of implants, which were not accounted for in this simulation. The study did not account for potential 3D effects of osteotomies, which could lead to different clinical outcomes. Radiographic projections and rotational deformities inherent to 2D X‐rays may influence the measurements, an issue that would be mitigated with a 3D approach.

Since the mathematical model relies on isosceles triangles, the conclusions apply solely to open wedge osteotomies. The study is also limited by the use of a single rater and the fact that it was conducted at only one centre, which may introduce bias and restrict the generalizability of the findings. Finally, it should be noted that this was a purely simulation‐based study, and the osteotomies were not physically performed.

This study emphasizes the importance of aligning preoperative planning with intraoperative execution in open wedge osteotomies, as deviations in hinge and entry points can lead to changes in wedge height and potentially affect the correction outcome. By measuring osteotomy depth intraoperatively and recalculating wedge height using the provided correction table (Table 1), surgeons could possibly adapt to deviations and achieve precise corrections, improving surgical accuracy and patient outcomes.

## CONCLUSION

This study highlights that relying solely on wedge height for osteotomy planning in HTO and DFO is insufficient due to variations in entry and hinge points. The hinge angle proved to be the most reliable parameter. Intraoperative osteotomy depth measurements can help adjust wedge height for accurate limb alignment when deviations occur.

## AUTHOR CONTRIBUTIONS

Julius Watrinet and Armin Runer designed the study. Johannes Schlaich collected data. Julius Watrinet and Romed Vieider performed the statistical analysis. Julius Watrinet, Marco‐Christopher Rupp and Armin Runer wrote the manuscript. Julian Mehl and Sebastian Siebenlist assisted with data interpretation and critically reviewed the manuscript. All authors read and approved the final manuscript.

## CONFLICT OF INTEREST STATEMENT

Julian Mehl is a consultant for Arthrex GmbH and Ormed GmbH. Sebastian Siebenlist is a consultant for Arthrex GmbH, KLS Martin Group and medi GmbH & Co. KG. The remaining authors declare no conflicts of interest.

## ETHICS STATEMENT

Ethical approval was given by the local ethics committee of the Technical University Munich (2024‐318‐S‐CB). This is a retrospective study. All patient information was deidentified and patient consent was not required.

## Data Availability

The data that support the findings of this study are available from the corresponding author upon reasonable request.

## APPENDIX A

**Table A1:** High tibial osteotomy value of heights and depths for each combination of osteotomy entry (A–C) and hinge point (1–9).

	Wedge height			Osteotomy depth		
	A	B	C	A	B	C
1	4.8 (4.2–5.7)	5.0 (4.2–5.7)	5.8 (4.8–6.4)	47.7 (47.1–51.6)	49.3 (46.7–51.5)	56.1 (53.4–57.8)
2	5.3 (4.8–6.4)	5.5 (4.7–6.4)	6.2 (5.2–7.1)	55.5 (53.4–59)	55.1 (53.1–58)	61.2 (58.5–63.4)
3	6.1 (5.4–7.3)	6.2 (5.3–7.2)	6.8 (5.7–7.8)	62.5 (59.9–66.4)	61.5 (59.1–64.8)	67 (63.9–69.5)
4	4.7 (4.1–5.6)	4.7 (3.9–5.5)	5.2 (4.4–5.9)	45.5 (44.8–49.6)	45.3 (42.7–47.6)	51 (48.4–52.4)
5	5.3 (4.7–6.5)	5.2 (4.6–6.2)	5.8 (4.9–6.6)	53.2 (51.4–57.2)	51.5 (49.8–54.6)	56.7 (54.2–58.3)
6	6.0 (5.3–7.3)	5.9 (5.1–6.8)	6.4 (5.4–7.3)	60.9 (58.1–64.9)	58.3 (56.1–61.8)	62.6 (59.8–65)
7	4.7 (4.1–5.6)	4.4 (3.8–5.1)	4.9 (4.1–5.6)	44.5 (43.6–48.5)	42.4 (39.8–44.8)	46.4 (43.7–47.8)
8	5.4 (4.7–6.6)	5.0 (4.4–5.9)	5.6 (4.6–6.3)	53.1 (50.5–56.5)	48.6 (47.2–51.8)	52.7 (49.9–54.3)
9	6.0 (5.4–7.5)	5.8 (5.0–6.8)	6.1 (5.2–7.1)	58.9 (57.1–63.7)	55.8 (53.8–59.4)	58.9 (56.2–61.2)

*Note*: Median (*Q*1–*Q*3).

**Table A2:** Distal femur osteotomy value of heights and depths for each combination of osteotomy entry (A–C) and hinge point (1–9).

	Wedge height			Osteotomy depth		
	A	B	C	A	B	C
1	4.2 (3.6–5.1)	3.9 (3.5–4.8)	4.0 (3.7–4.9)	42.2 (38.8–46)	39.7 (36.6–43.1)	42.2 (38–45.5)
2	4.4 (3.8–5.5)	4.0 (3.6–4.9)	4.0 (3.7–4.8)	45.6 (42–49.5)	41.8 (38.3–45.6)	42.3 (38.1–45.6)
3	5.1 (4.4–6.3)	4.8 (4.2–5.8)	4.8 (4.4–5.7)	52.5 (48.3–56.9)	49.5 (45.4–53.4)	50.2 (46–54.7)
4	4.7 (4.1–5.9)	4.2 (3.7–5.1)	4.0 (3.7–4.9)	49.7 (45.6–53.5)	44.3 (40.9–48.5)	43.2 (39.3–46.8)
5	5.4 (4.6–6.6)	4.9 (4.3–6.0)	4.8 (4.4–5.8)	56.2 (51.6–60.6)	52 (47.6–56.1)	51 (47.1–55.7)
6	6.0 (5.2–7.4)	5.7 (5.0–6.9)	5.7 (5.2–6.7)	63.1 (57.9–67.7)	59.7 (55.6–64)	59 (55.1–64.4)
7	5.6 (4.8–6.8)	5.2 (4.6–6.3)	5.2 (4.7–6.2)	57.9 (53.1–62.2)	54.7 (50–58.6)	54.5 (50.7 –59.5)

*Note*: Median (*Q*1–*Q*3).
